# The Subcellular Location of Selenoproteins and the Impact on Their Function

**DOI:** 10.3390/nu7053938

**Published:** 2015-05-22

**Authors:** Alan M. Diamond

**Affiliations:** Department of Pathology, University of Illinois at Chicago, Chicago, IL 60612, USA; E-Mail: adiamond@uic.edu; Tel.: +1-312-413-8747; Fax: +1-312-996-7586

**Keywords:** selenium, selenoproteins, peroxidases, compartmentalization, cancer

## Abstract

Most human selenium containing proteins contain selenium in the form of the amino acid selenocysteine, which is encoded in the corresponding mRNA as a UGA codon. Only a few non-selenocysteine containing selenoproteins are present and the nature of the association with selenium is not well understood. This review focuses on two selenocysteine-containing proteins that are members of the glutathione peroxidase family, GPx-1 and GPx-4, and the selenium-associated protein referred to as Selenium Binding Protein 1. Each of these proteins have been described to reside in two or more cellular compartments, and in the case of GPx-1 and SBP1, interact with each other. The enzymatic activity of GPx-1 and GPx-4 have been well described, but it is less clear how their cellular location impacts the health related phenotypes associated with activities, while no catalytic function is assigned to SBP1. The distribution of these proteins is presented as is the possible consequences of that compartmentalization.

## 1. Selenoproteins

Selenoproteins are a class of peptides that contain one or more atoms of selenium in tight association. The sub-class containing most of these proteins, and also the best characterized, includes those proteins in which selenium is present as the amino acid selenocysteine [1,2]. Selenocysteine is encoded by the UGA codon that directs the translational decoding of UGA codons rather than being used as a translational terminator if the corresponding mRNA includes a Selenocysteine Insertion Sequence (SECIS), which in eukaryotes, is present in the 3′-untranslated region (UTR) of the RNA [3,4]. The SECIS element, along with a host of dedicated translation factors that support the synthesis of these proteins is required for successful insertion of selenocysteine and this process has been reviewed extensively by others [5,6]. The other much smaller and less characterized family of selenium-containing proteins includes those in which selenium is tightly bound but do not contain a UGA-encoded selenocysteine [7].

Selenium containing proteins were originally detected by their ability to be labeled with radioactive Se^75^ [8]. In the case of selenocysteine-containing proteins, their identification was greatly facilitated by the development of an algorithm that could scan the sequence of entire genomes and detect genes that contained the hallmarks of selenocysteine encoding genes, including the in-frame UGA codon and sequences in the 3′-UTR characteristic of the SECIS element [9]. This approach has likely identified most and probably all of the proteins in this family for a particular species, with 25 selenoproteins being identified in humans. The biochemical function of most of these human selenoproteins has been determined by a variety of approaches. For many of these proteins, function was inferred based on predicted structure, the presence of sequence motifs linked to function, cell free assays and/or expression patterns and further investigated by altering their levels in cultured cells or animals. More difficult has been the assessment of the biological consequences of some of these proteins past the knowledge of their substrates and the chemical reactions they catalyze. In the case of non-selenocysteine encoding selenoproteins such as selenium binding protein 1 (SBP1), understanding their biological roles is even more difficult given how little is known about that protein’s functions in the cell.

## 2. Subcellular Location of Proteins Affects Their Biological Functions

The distribution of proteins in cellular compartments has long been recognized as having an impact on their function. One of the simplest and best studied scenarios is when a membrane bound receptor binds its ligand, resulting in its translocation to the cytoplasm where the receptor can interact with its substrate. Many translocated receptors eventually find their way into the nucleus where they engage binding sites in the genes they regulate and act as transcription factors to stimulate or repress expression. In other examples involving transcription factors, some are retained in the cytoplasm by interaction with a repressor. For example, the NFκB transcription factor is retained in the cytoplasm by interaction with it its repressor, IκB (inhibitor of IB) which masks the NFκB nuclear localization sequence, resulting in its sequestration outside of the nucleus and thus making it inactive [10,11]. Rapid activation of NFκB can be achieved by the post-translational modification of the bound IκB which results in its subsequent degradation, permitting NFκB to enter the nucleus and induce expression of a variety genes involved in the cellular response to stress. While none of the known selenoproteins have been demonstrated to be transcription factors, a better comparison might be the PTEN tumor suppressor protein. In the cytoplasm, PTEN removes a phosphate from phosphatidyl-3,4,5-bisphosphate (PIP_3_) and other protein substrates, resulting in the inhibition of the cell proliferation and the inhibition of apoptosis. A nuclear version of PTEN is believed to act on a completely different set of substrates resulting in enhanced DNA damage repair, centromere stability and apoptosis [12,13]. It is interesting that there are other examples of reactive oxygen (ROS)-sensitive or -responsive proteins in addition to NFkB and several of the selenoproteins where their cellular location impacts their functions. These include the Nrf2-Keap1 signaling pathway where the Nfrf2 transcription factor is retained in the cytoplasm until it dissociates from the Keap1 binding protein [14,15] and the HIF1α hypoxia-inducible transcription factor that accumulates in the nucleus when its degradation is inhibited under conditions of low oxygen tension [16,17]. The focus of this manuscript is the potential importance of the cellular localization of several selenoproteins on their functions.

## 3. GPx-4

Perhaps the most dramatic and best understood example of the differential partitioning of a selenoprotein in the cell is in the case of glutathione peroxidase 4 (GPx-4), also referred to as the phospholipid hydroperoxide glutathione peroxidase. GPx-4 is unique among the glutathione peroxidase family of proteins in that it can use reducing equivalents obtained from glutathione to reduce phospholipid hydroperoxides in the membrane [18,19]. The biological role of GPx-4 has been extensively reviewed [20,21,22]. Several different mRNAs transcribed from the same GPx-4 gene result in different forms of the protein that localize to either the cytoplasm, the mitochondria or the nucleus. The longest transcript, that which initiates from the most 5′ promoter and utilizes the first in-frame AUG start codon, contains a mitochondrial leader sequence (MLS) that directs the protein to the mitochondria where the leader is cleaved [23]. This form of GPx-4 is only expressed in testicular cells [24] where is essential for the proper formation of the sperm capsule [25,26] An alternative and shorter transcript is expressed from a downstream promoter which is translatated using a start codon that results in the omission of the MLS and a ubiquitiously expressed form of GPx-4 [24,27] and is likely to account for the majority of anti-oxidant functions described for this protein. This protein is located in the cytoplasm, nucleus and mitochondria of somatic cells and is essential for the formation of viable embryos [28]. An additional transcript exclusively expressed in spermatids is generated from a promoter located in the first intron of the GPx-4 gene [29,30]. The resulting protein functions in stabilizing chromatin in complex with protamines [31,32,33].

The unique roles of GPx-4 isoforms in mouse development have been examined by knocking down these different GPx-4 isoforms in cultured mouse embryos. While GPx-4 knockout mice are embryonic lethal, animals engineered to only express the cytoplasmic form are viable but sterile [28]. The impediment to embryonic development observed in the GPx-4 knockout mice cannot be rescued by the mitochondrial form [28,34]. Knocking out only the nuclear GPx-4 version results in only minor effects on chromatin condensation while maintaining fertility [31,33]. These elegant studies have clearly defined distinct roles for the three different GPx-4 proteins generated from the single corresponding gene.

## 4. GPx-1

The first characterized and best studied Sec-containing protein is the ubiquitously expressed glutathione peroxidase-1 (GPx-1). This enzyme uses reducing equivalents from glutathione to detoxify lipid and hydrogen peroxides, its levels are sensitive to selenium availability (see reference [35] for and extensive review) and has been reported to be localized to both the cytoplasm and mitochondria [36,37,38]. The possible functional significance of the distribution of GPx-1 between the mitochondria and cytoplasm was revealed by a recent study in which GPx-1 allelic variants were exclusively expressed in MCF-7 human breast cancer cells [39]. Two genetic variations in the human GPx-1 gene, a variable number of alanine-encoding triplets in the 5′- end of the gene and either a codon for alanine or proline at position 198 have been described and these have been shown to associated with several diseases including cancer (reviewed in [40]). The functionality of these variations was established by ectopically expressing individual GPx-1 alleles in MCF-7 cells which otherwise do not produce detectable GPx-1 mRNA or protein, and demonstrating a differing response to selenium supplementation to the media based on genotype [40,41]. Using the same approach, the expression of different GPx-1 alleles in MCF-7 cells, it was determined that there was a difference in the distribution of GPx-1 between the cytoplasm and the mitochondria with the allele containing a leucine at codon 198 and encoding 7 alanines be located more cytoplasmically than the allele with a corresponding proline and 5 alanines. In order to assess the significance of this cellular distribution, GPx-1 was engineered to exclusively partition to the mitochondria by engineering the protein to include a mitochondrial targeting sequence. Based on these data, it was concluded that the cellular distribution of GPx-1 impacted the levels of reactive oxygen species, the relative use of glycolysis *vs.* oxidative phosphorylation, the levels of the redox-sensitive transcription factor NF-κB and the phosphorylation of AKT, a serine-threonine protein kinase that plays a role in a host of vital cellular processes [39]. While the effects of directing GPx-1 to the mitochondria might be considered non-physiological due to the relatively large levels of GPx-1 in the mitochondria of transfected cells, differences in the molecular changes that resulted from the distinct GPx-1 proteins despite being expressed at similar levels are consistent with both cellular location and primary structure being consequential for GPx-1 function.

How the distribution of GPx-1 between the cytoplasm and mitochondria impacts biological processes is unknown. One possibility is that mitochondrially located GPx-1 is susceptible to post-translational modifications that can only occur in that organelle. For example, several reports have provided evidence that GPx-1 is a substrate for the SIRT3 mitochondrial deacetylase [42,43,44] and it’s localization to the cytoplasm may make it unavailable for modification. GPx-1 also interacts with the cytoplasmic Abl/Arg tyrosine kinase [45] and this is unlikely to occur when GPx-1 is in the mitochondria. One intriguing possibility is that mitochondrially located GPx-1 can efficiently reduce hydrogen peroxide generated from the dismutation of superoxide produced by electron transport and reduced by MnSOD. The importance of the removal of MnSOD generated hydrogen peroxide on energy metabolism and cellular signaling has recently been expanded upon [46] and the interaction between the genetic variations discussed above in the GPx-1 gene and polymorphisms in the MnSOD gene that impact breast cancer susceptibly has been reported [47].

GPx-1 compartmentalization also impacted the levels of Selenium Binding Protein 1 (SBP1), a non-selenocysteine containing selenoprotein whose levels have shown to predict the outcome of patients suffering from several different cancer types and for which there are data of a direct physical interaction with GPx-1 [7,48]. Additional details about SBP1 and its interaction with GPx-1 will be presented below.

## 5. SBP1

Selenium-Binding Protein 1 (SBP1, and also referred to as SELENBP1, hSP56) is a non-selenocysteine containing protein that forms a tight association with selenium, originally identified as a mouse protein that bound radioactive ^75^Se [49,50]. While the function of SBP1 is still unknown, altering its levels in a variety of tumor cells causes changes in several parameters associated with cellular transformation, including proliferation, senescence, and colony formation in semi-solid media [51,52,53,54,55,56]. Some of these phenotypes may be the result of the reported interactions with the von Hippel-Landau protein interacting deubiquitinating enzyme 1 [57] and the consequential effects on the levels of the HIF-1αtranscription factor that impacts the expression of multiple pathways in response to hypoxia [56]. Reduced levels of SBP1 have been frequently detected in tumors of a wide variety as compared to the corresponding normal tissues, and low SBP1 levels in tumors have also been shown to be an indicator of poor clinical outcome (reviewed in [7,58]).

SBP1 has been reported to reside in both the nucleus and the cytoplasm in tissues of several types, including lung adenocarcinomas [59], gastric adenocarcinomas [60,61] and both normal and malignant prostatic tissue [62]. Using human hepatocellular carcinoma cell lines, SBP1 was shown by Huang *et al.* to be localized to both the nucleus and cytoplasm while GPx-1 was exclusively cytoplasmic [52]. However, oxidative challenge to these cells with 50 mM hydrogen peroxide resulted in the co-localization of both proteins in the nucleus, and the authors suggested that this observation provided an indication that GPx-1 and SBP1 were physically interacting [52]. This notion is supported by *in vitro* studies where co-immunoprecipitation of these proteins was reported. In these studies, increasing the levels of SBP1 reduced GPx-1 enzyme activity but not mRNA levels [48] and reducing SBP1 levels using siRNA increased GPx-1 enzyme activity. Increasing GPx-1 levels and consequently enzyme activity led to a reduction in SBP1 levels [48]. Moreover, increasing or decreasing the levels of GPx-1 in colonic epithelial cells of mice by feeding a selenium-deficient, -adequate or -supplemented diet (0, 0.1 or 0.4 ppm selenium in the form of sodium selenite, respectively) resulted in an opposing effect on SBP1 levels [48]. The inverse association between GPx-1 and SBP1 levels was also observed in human prostatic tissue [63]. Given the physical interaction between SBP1 and GPx-1, it is possible that their association serves to sequester the other protein in one compartment which may either keep it in proximity to its substrate or prevent that from occurring.

**Figure 1:**
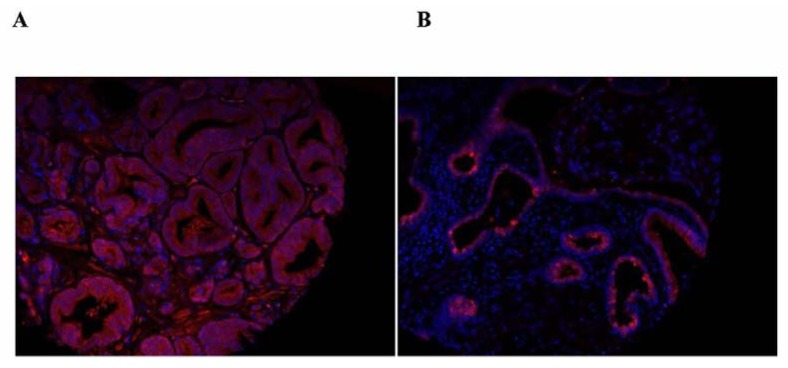
Samples of the images obtained upon staining human prostate tissue obtained from the outcome CPCTR tissue microarray with anti-SBP1 antibodies, shown in red. Nuclei are stained with DAPI which appears as blue. Panel **A** shows mostly cytoplasmic staining while panel **B** is an example of a tissue core that displays sporadic nuclear SBP1 staining.

The clinical significance of the distribution of SBP1 between the nucleus and the cytoplasm was recently expanded upon by quantifying SBP1 in the nucleus and the cytoplasm using a tissue microarray representing 202 prostate cancer patients whose cancer recurred after prostatectomy and 202 matched control tissue cores from patients whose disease did not recur [62]. Analysis of these samples indicated both nuclear SBP1 levels and the nuclear to cytoplasmic ratio of SBP1 inversely correlated with tumor grade. Examples of human images indicating the nuclear localization and the predominantly cytoplasmic location of SBP1 are presented in Figure 1. Knowledge of whether prostate cancer will recur or not is a challenging issue since many men are treated for indolent prostate cancer that is better off not treated. It is therefore potentially highly significant that patients whose tumor tissue was in the lowest quartile of nuclear SBP1 expression were significantly more likely to recur, as indicated by rising levels of prostate specific antigen (PSA), than patients with higher nuclear SBP1 [62]. Apart from the potential predictive value of nuclear SBP1 levels, these data may indicate that SBP1 cellular location may impact tumor biology, perhaps by shifting its availability to interact with other proteins or by some yet to be discovered enzymatic activity.

## 6. Thioredoxin Reductases

Mammalian thioredoxin reductases (TRs) are a family of selenium-containing proteins in which selenium is present as selenocysteine [64,65,66]. Thioredoxin reductase 1 and 2 are mostly cytoplasmic and mostly mitochondrial, respectively [66,67]. A third member and least characterized of the TR family, thioredoxin reductase 3, is involved in sperm maturation [68]. The functions of the TR family of proteins has been reviewed extensively [69,70,71] and won’t be discussed further here.

## 7. Conclusions

The cellular distribution of the selenoproteins discussed above is likely to have a profound impact on their functions and relevancy to human disease. This is already well established for GPx-4 but not so for GPx-1 and SBP1. The association between allelic variations in the gene that encodes GPx-1 with the risk of diseases and the partitioning between the cytoplasm and the mitochondria raises the possibility that this phenomenon reveals a critical and little understood function for this protein above what is already appreciated based on its known enzymatic function. In contrast, no enzymatic function has yet been attributed to SBP1, yet its lower levels in tumors and the association between its lower levels and the poor prognosis for cancer patients would indicate that this protein may eventually have predictive or therapeutic utility. Clues regarding SBP1 function may be derived from the observation that the protein is translocated to the nucleus when cells are subjected to oxidative stress and that the distribution between the nucleus and the cytoplasm is associated with prostate cancer aggressiveness. Whether the interaction between GPx-1 and SBP1 impacts their function or has a contributing role in cancer and other diseases remains to be determined.
